# Factors influencing an intention to use intrauterine device among family planning users in Southwestern Ethiopia

**DOI:** 10.1017/S1463423626101182

**Published:** 2026-04-27

**Authors:** Afework Tadele, Zerhun Asefa, Alemi Kebede, Tekle Wakjira, Demisew Amenu

**Affiliations:** 1 Department of Population and Family Health, Jimma University, Jimma, Ethiopia; 2 Department of Gynecology and Obstetrics, Jimma University, Jimma, Ethiopia

**Keywords:** family planning users, intention, intrauterine device, Southwest Ethiopia

## Abstract

**Introduction:**

An intrauterine device (IUD) is a highly effective long-acting and reversible contraceptive method widely available around the world and safe for nearly all women. However, very few women in Southwestern Ethiopia use.

**Objectives:**

To identify factors influencing an intention to use the intrauterine device among family planning users in Southwestern, Ethiopia.

**Methods:**

A facility-based cross-sectional study was conducted among 784 modern family planning users from 15^th^ October to 15^th^ November 2020. An interviewer-administered questionnaire was used. Estimates were generated using logistic regression model.

**Results:**

Thirty percent intended to use IUD. The most commonly cited reasons for their lack of an intention to use IUD were fear of side effects, lack of knowledge, and husband disapproval. Being able to read and write (AOR = 3.33 [95% C.I. 1.48, 7.49]) compared to those unable to read and write; Being rich (AOR = 1.69 [95% C.I. 1.02, 2.82]) compared to the poor; being knowledgeable about IUD (AOR = 2.74 [95% C.I. [1.67, 4.51]); having higher reproductive health autonomy (AOR = 1.53 [95% C.I. [1.09, 2.16]) were found to be significant factors influencing women’s intention to use an IUD.

**Conclusion:**

Nearly one-third of women who attend health facilities are currently using contraception reported an intention to use an IUD in the future. Public health interventions should focus on the cited reasons reaching all the community in need, and give priority for women who unable read and write, and lowest wealth status. Further interventional studies were recommended to determine effective interventions to increase women’s intention to use intrauterine device.

## Introduction

The intrauterine device (IUD) is a small, flexible, T-shaped, plastic device that is inserted into a women’s uterus through her vagina and cervix by trained health staff, and contains copper or a hormone (World Health Organization 2018). The IUD is a highly effective long-acting and reversible contraceptive (LARC) that is widely available around the world and safe for nearly all women. The copper IUD is hormone-free, and its side effect profile differs from that of hormonal IUDs. While hormonal IUDs often affect menstrual bleeding and mood, the copper IUD’s primary side effects are related to heavier periods. It also lasts longer (up to 10 years) than hormonal IUDs (which typically last 3 to 5 years. (Miller ER 2013; USAID, 2014; Balde et al., 2017).

Worldwide, IUDs are the second most popular contraceptive method (14.3%) (Joshi et al., 2015, UNFPA, 2016). However, in sub-Saharan Africa (SSA), where the general contraceptive prevalence is lower than other regions, the contraceptive method mix is skewed toward short-acting methods. Short-acting methods constitute 82% of modern contraceptive use, while permanent and long-acting and reversible contraceptives (LARCs) methods are used very little, by comparison (UN, 2013). Short-term methods have higher failure rates due to inconsistent use, leading to unintended pregnancies. This explains the emphasis on Long-Acting Reversible Contraceptives (LARCs) like IUDs by Ministries of Health, as their effectiveness is less user-dependent (UN, 2016; UN, 2019, Bearak et al., 2020).

Among women obtaining contraception from health facilities, IUD use varies significantly by region: from 18.6% in Eastern and South-Eastern Asia (UN, 2019), to 27.8% in Tunisia, and 36.1% in Egypt (Buhling et al., 2014). This is in sharp contrast to Ethiopia, where – despite a near tripling of modern contraceptive prevalence among married women nationally (from 14% in 2005 to 41% in 2019) – IUD use remains low at 2% within the health system, ranging from 0% in the Somali region to 5.2% in Addis Ababa (USAID, 2012; CSA, 2019).

The Ethiopia Federal Ministry of Health has considered the important role of LARCs and permanent methods (PMs) and want 20% of family planning users to be using LARCs or PMs. But recent data shows that injectable contraceptives were popular (23%), followed by contraceptive implants (8%), while IUD utilization is only 2% (FMOH, 2011).

Moreover, previous work revealed that IUD use in the Southwestern was very low (Teshome *et al*., 2020; Woldeyohannes *et al*., 2022; Dubale and Tesfaw, 2025). However, study conducted on an intention to use an IUD and influencing factors was lacking. While the broader LARC/PM initiative includes various methods and clients, this study specifically focused on identifying factors that most influence women intention to use an IUD in Southwestern Ethiopia.

## Methods

### Study setting and period

This study was conducted in Gambela, Illubabor Zone, and Bench Sheko Zone, in Southwest Ethiopia. Gambela region comprises three administrative zones (Anuak, Nuer, and Majang), 12 districts, and one special District (Itang). The region has a total population of 463,000 (Central statistical Agency (CSA) 2019). Illubabor Zone has 14 districts and one city administration, with a total population of 968,303 (Illubabor zone Health Office, 2020). Benji Sheko has 6 districts and 2 city administrations with a total population of 639,629 (Benchi-Sheko Zone Health Office, 2020). Overall, the study area has a total population of 2,070,932. The study was conducted from October 15 to November 15, 2020.

### Study design and population

A cross-sectional study design was employed. Among women of reproductive age (15–49 years) visiting health facilities to obtain family planning services. Sample size was estimated using a single population proportion formula with the following assumptions was used. Desired precision (d) = 5%, design effect 2, confidence level = 95%, and proportion of women who an intention to use IUD in = 50%. We factored in 10% non-response rate, and calculated a minimum sample size of 844.

**Figure 1.**
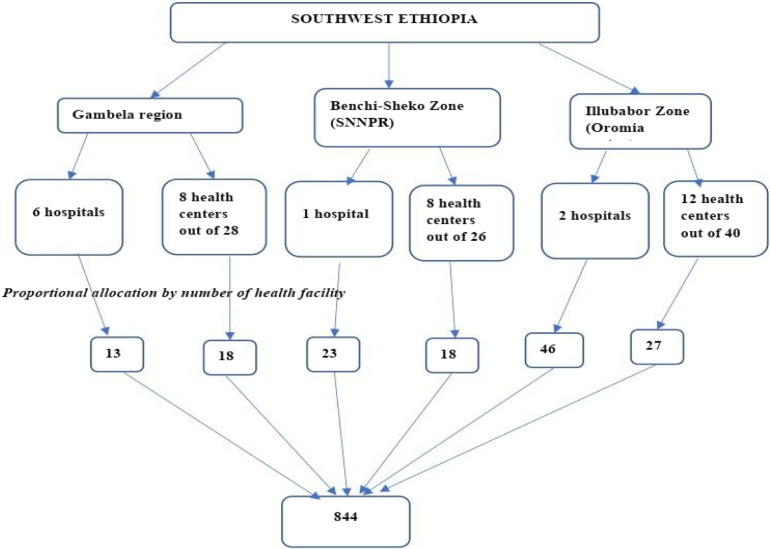
Schematic presentation of sampling techniques.

We selected all hospitals and 30% of the health centres available in the respective zones and region randomly by lottery method using simple random sampling (lottery draw from facility lists). Family planning clients were selected using systematic random sampling techniques after preparing sampling frame based on the preceding quarter report of each health facility using clinic attendance registers with sampling intervals calculated quarterly. A structured client exit interview administered questionnaire was used. The questionnaire was initially prepared in English, translated to local languages Afaan Oromo and Amharic, and back translated back to English for consistency. A pretest on 5% of the sample of women was conducted in Jimma Zone selected heath facilities (Figure 1).

### Data collection

Interviewer-administered questionnaires were conducted by trained nurses fluent in local languages. Before participation, written informed consent was obtained after explaining study objectives and confidentiality measures.

### Data management and quality

All data were electronically collected on-site and uploaded daily to the KoBo server using KoBocollect v1.25.1(kobotoolbox.org). Data were then exported to Stata 16.0 for further analysis. intention to use, misconceptions and utilization of IUD was the main outcome variable, whereas explanatory variables were: women’s socio-demographic, socio-economic characteristics, obstetric characteristics, and reproductive autonomy. We conducted descriptive analysis and bivariate and multivariable logistic regression to explore associations between intention to use IUD and selected independent variables. In bivariate logistics regression, a variable whose *p* < 0.25 was considered as a candidate for multivariate logistic regression analysis. And variables having *p* < 0.05 after multivariate logistic regression analysis were considered as independent predictors for intention to use IUD.

### Variable definitions and measurement

#### Intention to use IUD

Women who have a plan to use an IUD in the near future.

#### Knowledge about an IUD

Nine questions were asked about IUD and we classified based on the correct response to the questions. We used median to classify the study participants as knowledgeable and not knowledgeable, because the data does not fulfil normality assumptions. As such we dichotomized the participants as knowledgeable, if they correctly answered above median value, otherwise not knowledgeable.

#### Reproductive health autonomy

We used a validated tool to assess the reproductive health autonomy that comprised of three main domains: communication, decision making, and freedom from coercion (Ushma *et al*.,, 2014). After computing the index for the composite score, we classified as higher autonomy if they score above the mean value, otherwise lower autonomy.

#### Patient and public involvement

Patients participated as study respondents but were not involved in research design or analysis.

## Results

### Sociodemographic characteristics

A total of 784 women of reproductive age group participated in the study, yielding a response rate of 93%. The mean and standard deviation age of the study participants is 28 (+/−6) years. As shown in Table 1 and 4a clear distinctions between women with and without intention to use an IUD. Women intending to use an IUD were more likely to have higher educational attainment (51.6%, 95% CI [48.1%, 55.1%]) of degree holders intended to use an IUD vs. 48.4%, 95% CI [44.9%, 51.9]) who did not) and belong to a higher wealth category (38.2%, 95% CI [34.8%, 41.6%]) of the ‘Rich’ group intended to use an IUD). Strikingly, even basic literacy showed a strong association, with 47.2%, 95% CI [43.7%, 50.7%]) of women who could read and write intending to use an IUD. Significant ethnic variation was observed; for instance, 75.8%, 95% CI [72.8%, 78.8%]) of Nuer women intended to use an IUD, compared to only 12.0%, 95% CI [9.7%, 14.3%]) of Agnuak women. Marital status also played a role, with single women showing a higher intention (56.0%, 95% CI [52.5%, 59.5%]) compared to married women (28.9%, 95% CI [25.7%, 32.1%]).

**Table 1.** Sociodemographic characteristics of the participants in Southwest Ethiopia

Variables	Categories	IUD utilization		
		No intention	Intention	*P*-value
Age Mean = 28 ± 6 years	15–19	33 (64.7%)	18 (35.3%)	0.094
	20–24	151 (72.6%)	57 (27.4%)	
	25–29	186 (74.7%)	63 (25.3%)	
	30–35	112 (64.4%)	62 (35.6%)	
	>35	66 (64.7%)	36 (35.3%)	
Ethnicity	Agnuak	81 (88.0%)	11 (12.0%)	<0.001
	Amhara	66 (58.9%)	46 (41.1%)	
	Bench	69 (82.1%)	15 (17.9%)	
	Kaffa	22 (73.3%)	8 (26.7%)	
	Kanbata	13 (44.8%)	16 (55.2%)	
	Nuer	8 (24.2%)	25 (75.8%)	
	Oromo	254 (72.4%)	97 (27.6%)	
	Others	35 (66.0%)	18 (34.0%)	
Marital status	Married	522 (71.1%)	212 (28.9%)	0.010
	Separated (not officially divorced)	11 (68.8%)	5 (31.3%)	
	Single	11 (44.0%)	14 (56.0%)	
	Widowed	4 (44.4%)	5 (55.6%)	
Religion	Muslim	105 (71.9%)	41 (28.1%)	0.49
	Orthodox	124 (66.3%)	63 (33.7%)	
	Others	2 (50.0%)	2 (50.0%)	
	Protestant	317 (70.9%)	130 (29.1%)	
Educational status	Able to read and write	120 (81.1%)	28 (18.9%)	<0.001
	Diploma	19 (52.8%)	17 (47.2%)	
	Primary school (1–8 Grade)	166 (71.9%)	65 (28.1%)	
	Secondary school (9–12 Grade)	130 (65.0%)	70 (35.0%)	
	Unable to read and write	98 (71.0%)	40 (29.0%)	
	degree and above	15 (48.4%)	16 (51.6%)	
Respondent occupational status	Government employee	90 (63.8%)	51 (36.2%)	0.043
	Housewife	396 (73.1%)	146 (26.9%)	
	Merchant	36 (58.1%)	26 (41.9%)	
	Daily labour	4 (80.0%)	1 (20.0%)	
	Student	22 (64.7%)	12 (35.3%)	
Total		548 (69.8%)	236 (30.1%)	

### Obstetrics characteristics of study participants

There was no statistically significant difference in the intention to use an IUD across obstetric characteristics, although the proportions varied. The proportion of women intending to use an IUD was highest among those with two pregnancies (34.3%, 95% CI [27.2%, 42.2%]) compared to those with one pregnancy (24.8%, 95% CI [19.5%, 31.0%]) or more than three (30.0%, 95% CI [24.9%, 35.7%]). Experience with specific reproductive events also showed variation. Intention was somewhat higher among women who had ever encountered an abortion (33.8%, 95% CI [22.9%, 46.9%]) compared to those who had not (30.0%, 95% CI [26.5%, 33.7%]). In contrast, women who had never given birth showed the lowest level of intention (16.7%, 95% CI [4.7%, 44.8%]), though this estimate is based on a very small sample size and is thus highly uncertain. For other experiences, the intention to use an IUD was very similar. For example, the proportion among those who had an unintended pregnancy was 28.6% (95% CI [20.4%, 38.5%]) versus 29.5% (95% CI [26.0%, 33.3%]) among those who had not (Table 2).

**Table 2.** Obstetrics characteristics of study participants in Southwest Ethiopia

Variables	Categories	IUD utilization		*P*-value
		No intention	Intention	
Ever been pregnant	No	51 (70.8%)	21 (29.2%)	0.86
	Yes	497 (69.8%)	215 (30.2%)	
Number of pregnancies (*N* = 712)	one	173 (75.2%)	57 (24.8%)	0.11
	Two	113 (65.7%)	59 (34.3%)	
	More than three	217 (70.0%)	93 (30.0%)	
Ever encountered unintended pregnancy	No	423 (70.5%)	177 (29.5%)	0.84
	Yes	80 (71.4%)	32 (28.6%)	
Ever encountered abortion	No	452 (70.0%)	194 (30.0%)	0.096
	Yes	45 (66.2%)	23 (33.8%)	
Ever given birth	No	10 (83.3%)	2 (16.7%)	0.60
	Yes	493 (70.4%)	207 (29.6%)	
Ever encountered child/infant death	No	429 (70.3%)	181 (29.7%)	0.91
	Yes	63 (72.4%)	24 (27.6%)	
Total number of children (*N* = 698)	one	176 (74.3%)	61 (25.7%)	0.25
	Two	118 (67.4%)	57 (32.6%)	
	More than three	197 (68.9%)	89 (31.1%)	
Fertility status in the last five years	Not pregnant in the last five years	73 (67.0%)	36 (33.0%)	0.26
	Pregnant, Not wanted at All	11 (73.3%)	4 (26.7%)	
	Pregnant, wanted later	68 (68.7%)	31 (31.3%)	
	Pregnant, wanted then	342 (70.2%)	145 (29.8%)	
Total		548 (69.8%)	236 (30.1%)	

### Knowledge about IUD and level of reproductive health autonomy

Knowledge and exposure to information shows variation in an intention to use an IUD. Women who had heard of modern contraception were nearly three times more likely to intend to use an IUD (30.8%, 95% CI [27.5%, 34.3%]) compared to those who had not (11.1%, 95% CI [0.0%, 23.9%]), a difference that was statistically significant (*p* = 0.029). The source of information played a critical role. Intention to use an IUD was highest when information came from media sources 53.8% or health facilities 43.7%. Conversely, receiving information primarily from friends was associated with the lowest intention (15.4%, 95% CI [7.3%, 29.7%]). Most strikingly, being knowledgeable about the IUD was the strongest predictor. Among knowledgeable women, 41.1% (95% CI [36.5%, 45.9%]) intended to use it, compared to only 18.9% (95% CI [15.3%, 23.1%]) of non-knowledgeable women (*p* < 0.001). This represents more than a doubling of intention. Finally, women’s autonomy was examined in relation to IUD intention. Intention appeared higher among those with lower reproductive health autonomy (35.2%, 95% CI [29.6%, 41.3%]) compared with women reporting higher autonomy (27.4%, 95% CI [23.7%, 31.4%]) (*p* = 0.025). However, the overlapping confidence intervals indicate that this difference is not statistically significant and should be interpreted with caution (Table 3).

**Table 3.** Knowledge about IUD and level of reproductive health autonomy in Southwest Ethiopia

Variables	Categories	IUD utilization		*P*-value
		No intention	Intention	
Heard of modern contraception methods	No	24 (88.9%)	3 (11.1%)	0.029
	Yes	524 (69.2%)	233 (30.8%)	
Expose to IUD information	No	298 (79.3%)	78 (20.7%)	<0.001
	Yes	250 (61.3%)	158 (38.7%)	
Source of information about the about IUD (*N* = 408)	Friends	33 (84.6%)	6 (15.4%)	<0.001
	Health facility	80 (56.3%)	62 (43.7%)	
	Health facility friends	36 (76.6%)	11 (23.4%)	
	Health facility media	25 (51.0%)	24 (49.0%)	
	Health facility media friends	56 (64.4%)	31 (35.6%)	
	Media	18 (46.2%)	21 (53.8%)	
	Others	2 (40.0%)	3 (60.0%)	
Knowledgeable about IUD	Not knowledgeable	314 (81.1%)	73 (18.9%)	<0.001
	Knowledgeable	234 (58.9%)	163 (41.1%)	
Reproductive health autonomy	Lower	175 (64.8%)	95 (35.2%)	0.025
	Higher	373 (72.6%)	141 (27.4%)	
Total		548 (69.8%)	236 (30.1%)	

### Women’s intention to use IUD

**Figure 2.**
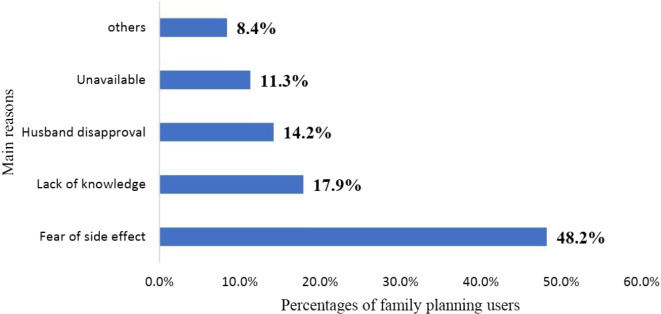
Main reasons for not intending to use an intrauterine device (IUD) in Southwest Ethiopia.

An intention to use IUD was 30.1% [95% C.I. 26.9%, 33.4%]. The most common cited reasons for not having an intention to use IUD were fear of side effect (48.2%), followed by lack of knowledge (17.9%), and husband disapproval (14.2%) (Figure 2).

### Factors influencing family planning user’s intention to use IUD

A multivariable logistic regression revealed that having an educational status, wealth status, being knowledgeable about IUD, and women’s reproductive health autonomy were found to be statistically Significant factors influencing an intention to use an IUD.

Statistically significant difference was observed even among mothers who never attended formal education being able to read and write was found to be more than three times more likely (AOR = 3.33 [95% C.I. 1.48, 7.49]) to have an intention to use IUD compared to those unable to read and write.

Although IUD was being provided for free in public health facilities significant difference was found between among rich and poor households. Being in the highest wealth status about twice (AOR = 1.69 [95% C.I. 1.02, 2.82]) more likely to have an intention to use IUD compared to those in the lowest wealth status.

Even after controlling for potential confounders being knowledgeable about IUD were more than three times (AOR = 2.74 [95% C.I. [1.67, 4.51]) more likely to have an intention to use IUD compared to their counterparts.

Furthermore, reproductive health autonomy was found to be statistically significant association. Having higher reproductive health autonomy was about twice (AOR = 1.53 [95% C.I. [1.09, 2.16]) more likely to have an intention to use IUD compared to those who have lower autonomous (Table 4).

**Table 4.** Multivariable logistic regression analysis of factors influencing family planning user’s intention to use IUD in Southwest Ethiopia

Factors	Categories	IUD utilization		COR [95% CI]	AOR [95% CI]	*P*-value
		No intention	Intention			
Educational level	Unable to read & write	120 (81.1%)	28 (18.9%)	1	1	
	Able to read & write	19 (52.8%)	17 (47.2%)	3.83 [1.77, 8.30]	3.33 [1.48, 7.49]	0.004
	Primary education	166 (71.9%)	65 (28.1%)	1.67 [1.01, 2.77]	1.12 [0.66, 1.91]	0.68
	Secondary education	130 (65.0%)	70 (35.0%)	2.30 [1.39, 3.81]	1.25 [0.69, 2.25]	0.457
	Diploma & above	113 (66.9%)	56 (33.1%)	2.12 [1.26, 3.58]	0.92 [0.48, 1.77]	0.804
wealth status	Poor	205 (78.2%)	57 (21.8%)	1	1	
	Average	183 (69.6%)	80 (30.4%)	1.57 [1.06, 2.33]	1.27 [0.82, 1.97]	0.278
	Rich	160 (61.8%)	99 (38.2%)	2.22[1.51, 3.27]	1.69 [1.02, 2.82]	0.043
IUD Information	Yes	298 (79.3%)	78 (20.7%)	2.41 [1.75, 3.32]	1.16 [0.71, 1.91]	0.145
	No	250 (61.3%)	158 (38.7%)	1		
Knowledgeable about IUD	Yes	314 (81.1%)	73 (18.9%)	2.99 [2.16, 4.14]	2.74 [1.67, 4.51]	0.000
	No	234 (58.9%)	163 (41.1%)	1	1	
Reproductive health autonomy	Lower	175 (64.8%)	95 (35.2%)	1		
	Higher	373 (72.6%)	141 (27.4%)	1.43 [1.05, 1.96]	1.53 [1.09, 2.16]	0.013
_cons					0.11 [0.05, 0.23]	0.000

## Discussion

Identifying that status and factors influencing family planning users’ intention to use an IUD is has of paramount importance for decision makers in knowing where the region is in achieving the global goals of maternal and child health, through addressing unmet need for family planning in contextualized approach.

In this study an intention to use IUD among family planning clients in the Southwestern Ethiopia was found to be thirty percent. This finding was higher than the study conducted in Debre Markos Town, North West Ethiopia (11.6%) (Bulto et al., 2014), and lower than the study from Nekemte town, Ethiopia (47.9%) (Tekelab et al., 2015). This difference may be due to the sampled populations and study settings, in this study two zones and Gambela region was included. Also, in both rural and urban populations were considered where only urban women were sampled in Debre Markos and Nekemte study.

The main reason listed for not using IUD includes lack of information, fear of side effect, not informed by health care providers, religion, husband disapproval, removal related issue, and misconceptions. This study was in line with the report from three African countries, Senegal, Mali, and Guinea where opposition from others were the most prevalent reason of nonuse of contraception. This finding is in line with a study conducted among the women of urban areas of Nepal (Joshi et al., 2014, Moreira et al., 2019). Furthermore, this study revealed that supply-side barriers for not using IUD were lack of training on IUD among health care providers. This finding is similar to a finding in Ghana (Robinson et al., 2016). This implies that even if the training was given by different stakeholders for health professionals, there is high staff turnover from time to time.

The study also revealed that women’s intention to use IUD were significantly influenced by wealth status. Women who live in lowest household wealth were less likely to have an intention to use IUD compared to women in the richest household wealth. This could be due to lack of information adequate information about the method they fear side effects and health seeking behaviour of this population is generally low.

In addition, this study revealed that attending formal education were found to be influence women’s intention to use IUD even after controlling for potential cofounder. Women who able to read and write were more likely to have an intention to use IUD compared to their those unable to read and write. This finding is supported by evidence from Uganda, and southern and central parts of Ethiopia (Wolaita Zone, and Mojo) (A. and N. 2011; Meskele and Mekonnen 2014; Twesigye et al., 2016). This might be due to women’s education helps to create more media exposures and have better knowledge of the family planning methods. In addition, this study, also showed that women’s knowledge about an IUD have an influence on their future intention to use it. This is consistent with study in Brazil that reported the level of knowledge about the intrauterine device was associated with the interest in using it (Borges *et al*., 2020). This implies, knowledge is basic for using the service.

Moreover, this study revealed that family planning users’ intention to use an IUD was found to be significantly influenced by women’s level of reproductive health autonomy. Women who have higher reproductive health autonomy have an intention to use IUD compared to those who have lower autonomy. Likewise, a cross sectional study in Mizan-Aman, South Ethiopia also reported that women who have higher decision-making power were more likely to use contraceptives (Belay *et al*., 2016). In addition, study in Democratic Republic of Congo showed that women’s autonomy as an important predictor of post-delivery modern contraceptive use (Sano *et al*., 2018). This implies that women reproductive health autonomy is critical in influencing their reproductive health goals.

### Strengths and limitations of this study

Strengths include high response rate (93%) and validated autonomy measures. Limitations include social desirability bias in spousal approval reporting and lack of qualitative data on cultural barriers. The cross-sectional design prevents causal inference. Only one third of family planning users has an intention to use of IUD in the future was very low, although family planning services were delivered for free at public health facilities in Ethiopia. This requires behaviour change communication among these segments of population. The most common reasons for not having an intention to use IUD were fear of side effects, lack of knowledge, and husband disapproval.

## Conclusion

This study concludes that intention to use an intrauterine device (IUD) is influenced by a complex interplay of factors beyond knowledge alone. While awareness of the method was a prerequisite, intention was significantly shaped by deeper socio-cultural determinants, including ethnic background and, most notably, spousal approval. A key finding was the positive association between literacy and IUD intention, suggesting that even basic literacy skills may enhance reproductive autonomy independent of formal education. The primary barriers identified were misconceptions about medical side effects, a simple lack of information, and partner disapproval. Therefore, public health interventions must adopt an integrated strategy that concurrently addresses community-level myths through targeted education, actively engages men as supportive partners in family planning, overcomes economic barriers, and strengthens provider competency in client-centred counselling. Future efforts should combine these supply-side and demand-generation approaches, and further interventional research is needed to determine the most effective strategies for increasing IUD uptake in this context.

## Data Availability

The STATA data used to support the findings of this study are available from the corresponding author upon request.
